# Supplementary material for: The Mechanism of Oxymatrine Targeting miR-27a-3p/PPAR-γ Signaling Pathway through m6A Modification to Regulate the Influence on Hemangioma Stem Cells on Propranolol Resistance
Source: Cancers (Basel). 2023 Oct 30;15(21):5213. doi: 10.3390/cancers15215213 (PMC10649746; doi:10.3390/cancers15215213)
Supplement: Supplementary file 1 [file cancers-15-05213-s001.zip › cancers-2553517-supplementary.pdf]

Supplementary Materials

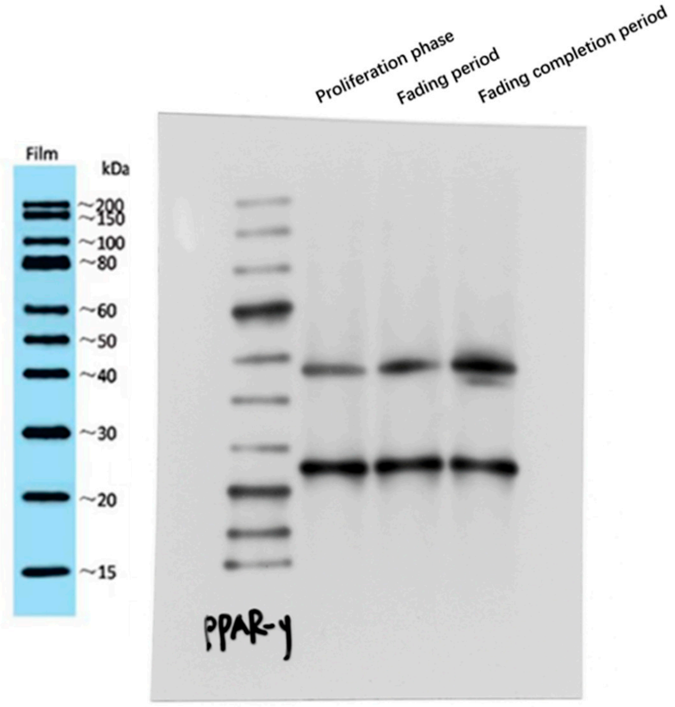

Supplementary Figure S1: The riginal protein blots of Figure 2B. The protein levels of PPAR-γ in the proliferation, regression, and completion stages of IH were determined using Western Blot.

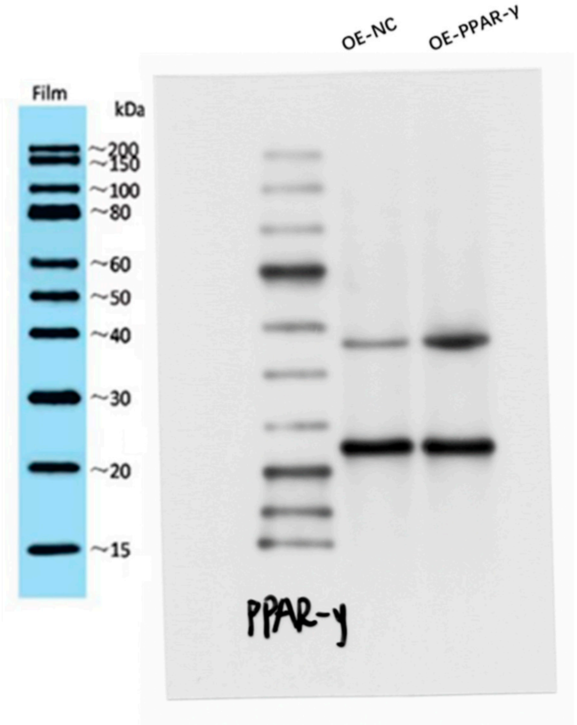

Supplementary Figure S2: The riginal protein blots of Figure 4A. The transfection efficiency of OE-PPAR-γ was assessed by Western Blot.

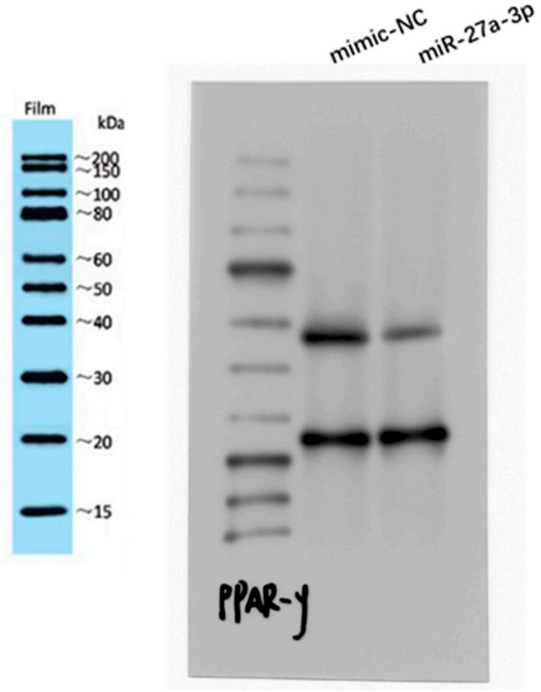

Supplementary Figure S3: The riginal protein blots of Figure 5E. Western Blot analysis were employed to examine the effects of miR-27a-3p on the expression and protein levels of PPAR-γ.

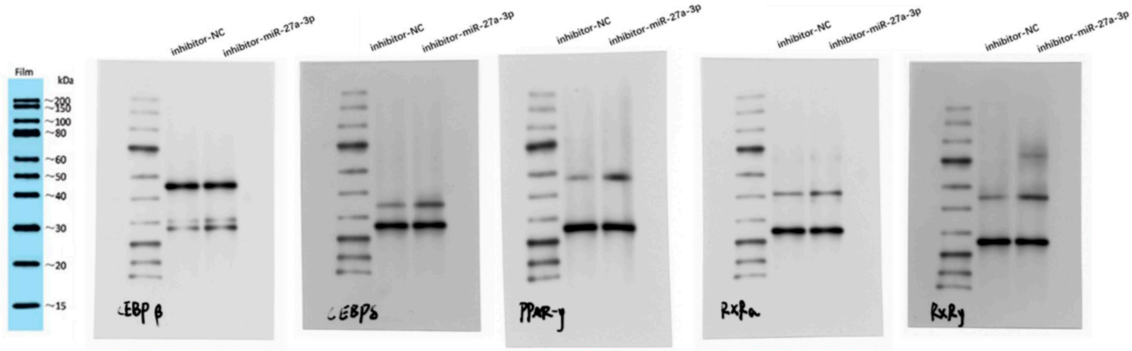

Supplementary Figure S4: The riginal protein blots of Figure 7C. Western Blot was used to detect the effect of miR-27a-3p-inhibitor.

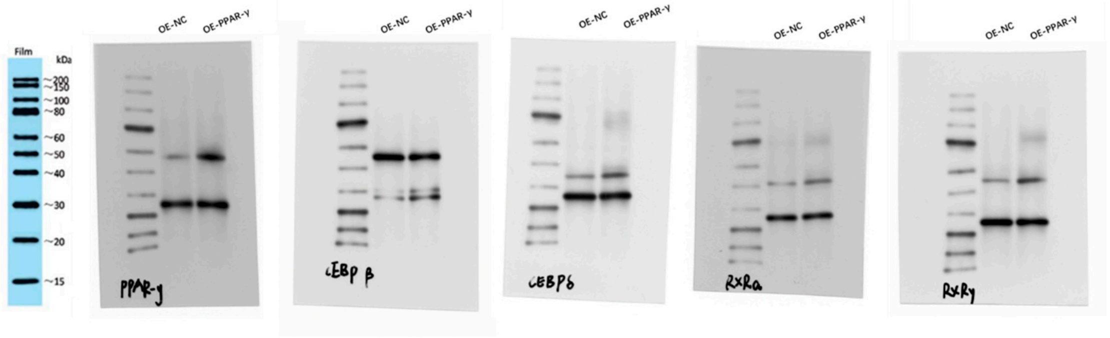

Supplementary Figure S5: The riginal protein blots of Figure 7D. OE-PPAR-γ and its effect on the expression of proteins related to adipogenesis in HemSCs, in-cluding PPAR-γ, C/EBPβ, C/EBPδ, RXRα, and RXRγ.

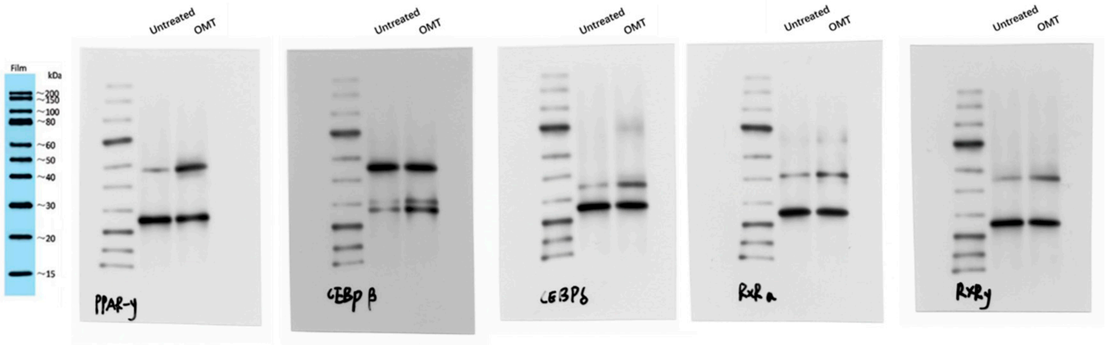

Supplementary Figure S6: The riginal protein blots of Figure 9B. Western Blot detected the effect of OMT on HemSCs cell fat differentiation-related proteins.

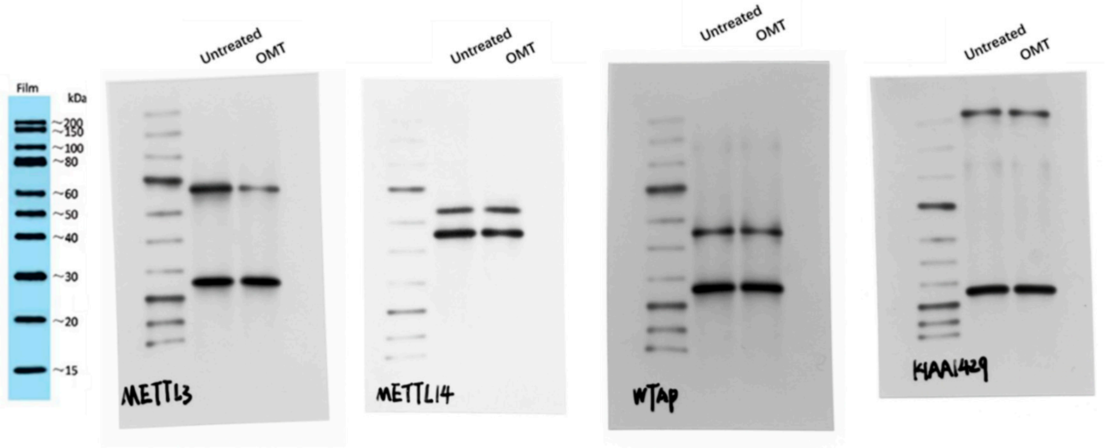

Supplementary Figure S7: The riginal protein blots of Figure 10A. Western Blot detects the effect of OMT treatment on the protein levels of HemSCs/PPNL drug-resistant cells METL3, METL14, WTAP and KIAA1429.

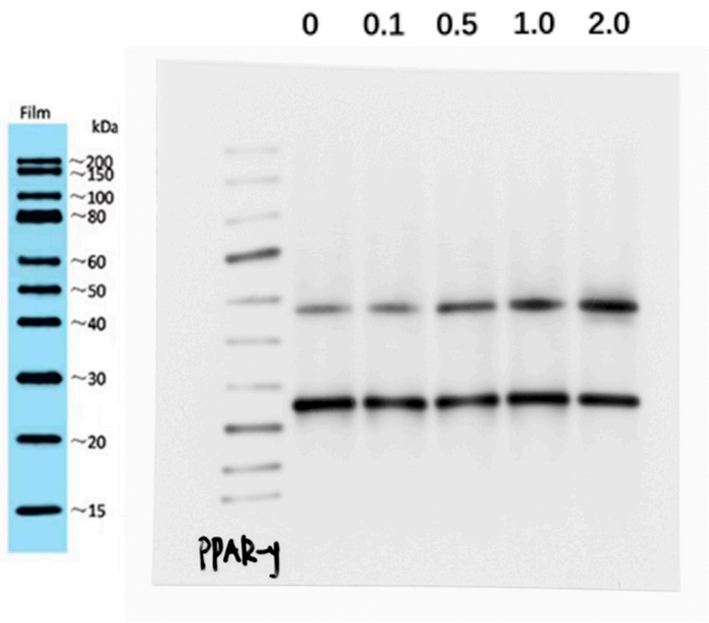

Supplementary Figure S8: The riginal protein blots of Figure 10F. Western Blot detection of the effect of OMT on HemSCs cell PPAR-γ protein level.

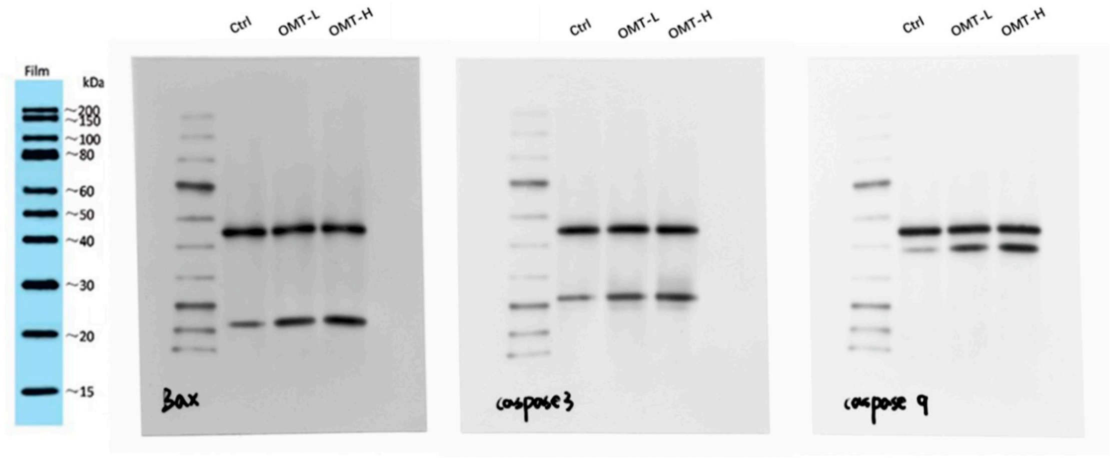

Supplementary Figure S9: The riginal protein blots of Figure 11D. Western Blot detection of apoptosis-related proteins under different treatments the protein expression of Bax, Caspase 3 and Caspase 9.
